# Efficacy of Brexpiprazol in treating psychotic symptoms and possible side effects

**DOI:** 10.1192/j.eurpsy.2026.12157

**Published:** 2026-07-31

**Authors:** A. M. Lazarraga, J. C. F. Talavera, G. G. Valera, G. L. Chapatte, M. R. Vaquero, L. R. Vazquez, A. Iglesias Navarro, M. M. Merino

**Affiliations:** 1 Psychiatry, Hospital Clinico de Valadolid, Valladolid; 2 Psychiatry, Hospital Principe de Asturias, Madrid, Spain

## Abstract

**Introduction:**

In the following study, we will try and evaluate the efficiency of Brexpiprazol in treating psychotic symptoms in ten patients with ages between 18-70 years old. We have researched and concluded there is scientific evidence that supports the use of Brexpiprazol to treat psychotic symptoms.

**Objectives:**

Our aim in this study is to try and evaluate the efficacy of Brexpiprazol (2-4mg) in treating psychotic symptoms and possible side effects. We have evaluated ten patients who have been taking brespiprazol and then evaluated with a Psychotic Symptom Rating Scales (PSYRATS), before and after two months of taking Brexpiprazol to try and see if it was effective for treating these symptoms. Meanwhile if any side effects were reported, they would be recorded (Drake, 2007)

**Methods:**

We will evaluate ten patients between the ages of eighteen and sixty-five before and after starting pharmacological treatment with brexpiprazole. At the beginning of treatment, we will use the PSYRATS scale to evaluate positive symptoms, like hallucinations and delusions. After two months, we will re-apply the scale to assess whether there has been remission of these symptoms.

**Results:**

We have evaluated 10 patients, five woman and five men, and we have introduced Brexpiprazol either 2mg, or 4mg in two doses, and evaluated their psychotic symptoms before using this drug and after two months of continuisly taking it. All ten patients had a significant decrease in their auditory hallucination scores, including duration, level of distress, and intensity.

The four patients that along with hallucinations had also delusions, their preoccupation and the intensity of which had decreased

and their life disruption had improved.

In total, there was a decrease of more that 50% of the total puntuation in PSYRATS within the ten evaluated patients. The reported side effects that subsided around the second week were drowsiness and sweating.

**Image 1:**
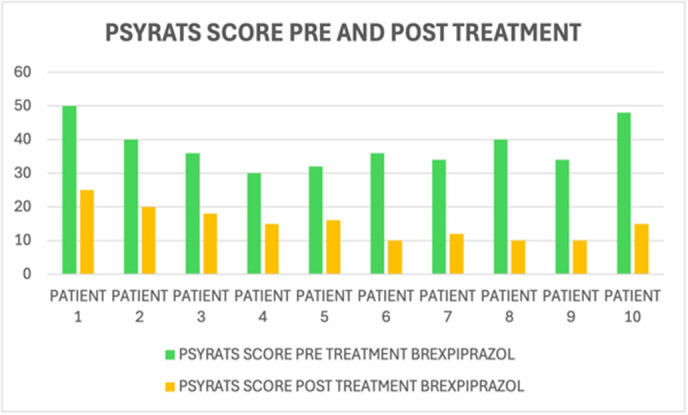
Image 1: Long description.

**Conclusions:**

In conclusion, in these ten patients who were treated with this drug at doses between 2 mg and 4 mg, we can affirm that what was observed was a decrease in hallucinations as well as delusions in those patients who maintained treatment for at least two months.It would be very interesting to be able to evaluate these patients after six months and one year, to be able to assess whether there continue to be a decrease of said symptoms as well as if there are any other side effects to be reported.

**Disclosure of Interest:**

None Declared

## Long descriptions

### Image 1: Long description

The chart displays P S Y R A T S scores on the Y-axis, ranging from 0 to 60 in increments of 10. The X-axis lists Patient 1 through Patient 10. Each patient has two bars: a green bar for pre-treatment with Brexpiprazole and a yellow bar for post-treatment.

* Patient 1: Pre 50, Post 25.

* Patient 2: Pre 40, Post 20.

* Patient 3: Pre 36, Post 18.

* Patient 4: Pre 30, Post 15.

* Patient 5: Pre 32, Post 16.

* Patient 6: Pre 36, Post 10.

* Patient 7: Pre 34, Post 12.

* Patient 8: Pre 40, Post 10.

* Patient 9: Pre 34, Post 10.

* Patient 10: Pre 48, Post 15.

Across all ten patients, the yellow post-treatment bars are significantly lower than the green pre-treatment bars, indicating a reduction in P S Y R A T S scores following the administration of Brexpiprazole.

Navigate back to Image 1:.

